# Salutatory

**Published:** 1860-09

**Authors:** J. G. Westmoreland


**Salutatory.**

In assuming the editorial control of this Journal the weight of responsibility connected with the position is not overlooked. The advocacy of correct principles in Journalism is not only important in directly tending to a healthy condition of the body of the profession, but in its ultimate tendencies redounds to the amelioration of the physical suffering ot our race. Tremblingly alive to these facts, and conscious of the want of ability for so important a trust, we make this our debut upon the stage editorial; pledging ourself sacredly to the profession, that to the utmost of our ability, what we have, what we are, and what we can do, shall be brought to bear in promoting the great cause of medical science, the best interest of the profession, and the general good of suffering humanity.

In this new field of action it will of course not be expected of me such proficiency as would, under other circumstances, be attainable. Not only so, but any failure to come up to the degree of perfection in the management of the work arrived at by the former editors should not be expected, for a while at least. And while every effort in our power will be made to render it, while in our charge, as useful and popular as possible, we would not flatter ourself, or deceive our readers with the promise that even an equal amount of talent editorial with that which was exercised in former volumes, will be found in this.

We ask, respectfully and earnestly, a continuance of the important aid rendered by valuable contributors to its pages, who have from the commencement of the publication, afforded, from time to time, valuable original articles. We hope not only that those who have heretofore rendered service to the cause of science and humanity, through the pages of this Journal, may continue to do so, but that others may be induced to devote an occasional hour in penning for publication such interesting facts as constantly come under their observation. We wish to appropriate a larger space than usual to original communications, in the form of re ports of cases, essays on medicinal agents and their application in the treatment of disease, pathology of disease, &c. With the country practitioner, whose business extends over so large a territory, and whose time is so nearly occupied with his professional duties, little time can be devoted to such labor; but without material detriment to themselves, interesting practical facts may be noted in a short article frequently, by those in the active duties of the profession, who, without any pretension to the claim of extensive writers, afford important new and practical truths for the benefit of the profession. With such aid from professional brethren we have no doubt of making it answer one of the important objects of Medical Journals—to serve as a medium of communication with each other, and as the means of conveying new practical facts and thoughts to each other. In such interchange of views, thought is excited, not only in him who reads, but in him who writes.

We repeat, that whatever of talent we may be able to bring into requisition, shall be exercised to the fullest extent in forwarding the cause of Medical Science, and in the promotion of the best interests of the profession. To these ends, investigation, in the yet uncertain theories, will be encouraged, and the thorough preparation of those asking admission into the great Medical family, will be zealously advocated. In seeking the accomplishment of the latter, no stab-in-the-dark movement will be tolerated, having ostensibly for its object the elevation of the standard of requirements, without one good reason in itself or one good motive in the mover, whether such emanate from a grade high or low, in the profession—in the most humble village society, or in the Medical Congress of the Union. While correct principles and truth will be supported to the utmost of our ability, the Journal will be a free medium of communication to all in the profession, and we solicit an interchange of opinion, side by side with ours, from those who may differ with us on any point connected with scientific subjects, or on questions involving the best interests of the profession.

While it has ever been our course, and the policy of the Journal, heretofore, to oppose anything sectional in Medicine, without good cause, as we would do in politics or religion, yet to the unreasonable and unjust oppression of minorities by sectional majorities, in the adoption of measures bearing unequally, without effecting any general good to the profession, we are, and have ever been, for uncompromising resistance. Reason shall direct us into the channel of justice and truth, in all things. With moderation and circumspection, yet with firmness and honesty, do we desire to deal with subjects proper for our consideration.

It is due the former Editor, to state that the confusion found to exist in the financial department of the Journal, is not attributable to any want of care on his part, during the short time he controlled its finances. The derangement in the subscription accounts existed prior to that time, and while that responsibility rested upon ourself. This, under the circumstances, however, was unavoidable. The names of subscribers, as well as payments for subscription, were received and entered by agents, by ourself and by the Editor. These irregularities, together with the transcribing in a new book, the names of subscribers, date of subscription, and amount of payments, have led to several errors already detected, and perhaps to others not yet discovered. Lest some may still exist, to the annoyance of our patrons, the former Editor, in sending out the amount of dues, addressed also a circular to each subscriber, asking him to correct any error he might find in his bill. This, we hope, will be done, and remittance (by mail, at our risk) of arrearages be made at once. Let every reader of the Journal remember that it requires an immense amount of mental and physical toil to get up the matter for such a periodical, and, in addition to this, for the Editor to shoulder the very respectable sum of twelve or fifteen hundred dollars during the year for its publication, makes it altogether important that the subscription price be paid, which, with the best possible collections, will not be sufficient to replace the actual outlay of money. The small amounts due from individual subscribers, might be remitted without detriment to any, and by so doing relieve us from responsibility truly embarrassing.

We know that delinquency on the part of our patrons is not to be attributed to a willful desire to withhold from us our dues, or that which is necessary to relieve us from actual pecuniary loss, but from experience we know that such obligations fail to be met, because subscribers are not usually impressed with the importance these sums are, in the aggregate, to the proprietors. Each reader of the Journal will be satisfied that the amount of his indebtedness is to that extent important to us, when he is reminded of the fact that the publisher has to be paid in *cash* every month, whether the Editors and Proprietors get that amount from the patrons or not.

The terms of the Journal require cash in advance for subscription, but in getting up a circulation of the Journal, it became necessary in many instances to vary from this rule. Many of these are now in arrears; with some of whom, no doubt, it is only required that we call their attention to our wants. Those who are not moved by our necessities, or who do not wish to continue the Journal at the price we are compelled to charge, will please notify us before our next issue.

J. G. WESTMORELAND.

